# Multi-Scale Permutation Entropy: A Potential Measure for the Impact of Sleep Medication on Brain Dynamics of Patients with Insomnia

**DOI:** 10.3390/e23091101

**Published:** 2021-08-25

**Authors:** Yanping Guo, Yingying Chen, Qianru Yang, Fengzhen Hou, Xinyu Liu, Yan Ma

**Affiliations:** 1 School of Electrical & Mechanical Engineering and Automation, Nanhang Jincheng College, Nanjing 211156, China; zhfy@nuaa.edu.cn; 2 School of Science, China Pharmaceutical University, Nanjing 210009, China; YingyingChen@njust.edu.cn (Y.C.); 2020170305@stu.cpu.edu.cn (Q.Y.); houfz@cpu.edu.cn (F.H.); 3 School of Software, Nanjing University of Science and Technology, Nanjing 210094, China; 4 Osher Center for Integrative Medicine, Brigham and Women’s Hospital and Harvard Medical School, Boston, MA 02215, USA; dr.yan.ma@gmail.com; 5 Beth Israel Deaconess Medical Center, Division of Interdisciplinary Medicine and Biotechnology, Department of Medicine, Harvard Medical School, Boston, MA 02215, USA

**Keywords:** permutation entropy, complexity, insomnia, EEG, temazepam

## Abstract

Insomnia is a common sleep disorder that is closely associated with the occurrence and deterioration of cardiovascular disease, depression and other diseases. The evaluation of pharmacological treatments for insomnia brings significant clinical implications. In this study, a total of 20 patients with mild insomnia and 75 healthy subjects as controls (HC) were included to explore alterations of electroencephalogram (EEG) complexity associated with insomnia and its pharmacological treatment by using multi-scale permutation entropy (MPE). All participants were recorded for two nights of polysomnography (PSG). The patients with mild insomnia received a placebo on the first night (Placebo) and temazepam on the second night (Temazepam), while the HCs had no sleep-related medication intake for either night. EEG recordings from each night were extracted and analyzed using MPE. The results showed that MPE decreased significantly from pre-lights-off to the period during sleep transition and then to the period after sleep onset, and also during the deepening of sleep stage in the HC group. Furthermore, results from the insomnia subjects showed that MPE values were significantly lower for the Temazepam night compared to MPE values for the Placebo night. Moreover, MPE values for the Temazepam night showed no correlation with age or gender. Our results indicated that EEG complexity, measured by MPE, may be utilized as an alternative approach to measure the impact of sleep medication on brain dynamics.

## 1. Introduction

Insomnia is a common sleep disorder that mainly manifests as difficulty falling asleep, difficulty maintaining sleep and frequent and early awakenings [1]. Insomnia negatively affects health and quality of life, and is strongly associated with the incidence and deterioration of diabetes [2,3,4], cardiovascular disease [5,6,7], cancer [8,9,10], cognitive impairment [11,12,13], brain damage [14,15] and depression [16,17]. In addition, blood circulation, endocrine dyscrasia, loss of immunological competence and thermoregulation are also affected by insomnia [18]. Prevalence of insomnia in the general population in China is about 15% [19] and insomnia has become the most commonly encountered sleep disorder in the United States, with prevalence rates of 15% to 24% [20].

Major categories of insomnia drugs include benzodiazepine receptor agonists, melatonin receptor agonists, histamine receptor antagonists and orexin/retinol receptor antagonists [21]. Temazepam is a type of benzodiazepine drug, which is the most widely used therapeutic drug to generate sedative, hypnotic and anti-anxiety effects for insomnia [22,23].

The current evaluation of insomnia drugs mainly relies on self-reported sleep quality using questionnaires, including the Pittsburgh Sleep Quality Index [24,25] and Insomnia Severity Index [26]. However, sleep misperceptions and subjective–objective sleep discrepancy have long been observed in patients with insomnia, and even in the general population [27,28,29]. Despite issues and debates on the use of polysomnography (PSG)-based sleep studies in insomnia, it is still the most commonly used objective evaluation for insomnia. Major sleep outcomes (e.g., number of awakenings, sleep onset latency, total sleep time, sleep efficiency, wake after sleep onset) are based on manual scoring on overnight recordings. Although these outcomes can be used to evaluate the effect of sleep drugs [30,31], negative factors of it being time-consuming, inter-scorer variability, the high demands of well-trained professionals and multiple channels of signals must also be considered. Therefore, the demand for alternative approaches for the evaluation of sleep associated with therapeutic interventions has never stopped.

In recent decades, complexity-based measurements have opened a new path to the quantification of the dynamic characteristics in physiological signals, and have been applied to sleep electroencephalogram (EEG) data to understand the complexity of brain activities during sleep [32]. Permutation Entropy (PE) is a common complexity measure that offers simple calculation and high robustness against noise [33]. In 2015, Deng et al. analyzed PE among patients with Alzheimer’s disease and showed that PE was an effective diagnostic index for patients with Alzheimer’s disease [34]. In 2016, Bian et al. analyzed PE among patients with type 2 diabetes and showed that PE was a potential indicator for diagnosing mild cognitive impairment to type 2 diabetes [35]. In 2017, Christoph et al. applied PE in sleep staging [36]. In 2018, multi-scale PE (MPE) was employed as a feature in the support of a vector machine model for seizure prediction by Yang et al. among patients with epilepsy [37]. Similarly, MPE was also adopted in the machine learning model aiming to provide an early diagnosis of autism by Zhao et al. in 2019 [38]. Recently, MPE was suggested as a potential indicator of sleep pressure for the general population [39].

In this paper, EEG MPE of healthy control subjects and patients with mild insomnia were analyzed to explore the value of MPE in the assessment of insomnia and temazepam treatment.

## 2. Methods

### 2.1. Participants

Two sleep datasets, Sleep Cassette Study (SC) and Sleep Telemetry Study (ST), were included in this study. Both datasets were obtained from an open-access database, namely the Sleep-EDF Database (Expanded) in PhysioNet [40,41]. The SC dataset included 78 healthy Caucasians and the ST dataset included 22 Caucasians who had mild difficulty falling asleep but were otherwise healthy. In the SC dataset, PSGs were recorded during two subsequent day–night periods at the participants’ homes, and these participants maintained their normal activities except for wearing a modified Walkman-like cassette-tape recorder, which has been described elsewhere [41]. Due to the failure of cassette or laser disk, 3 participants were excluded from the SC dataset. Therefore, our study included 75 participants (34 males, 59 ± 22 years old, mean ± standard derivation) from the SC dataset as healthy controls (HC). In the HC group, participants had no sleep-related medication intake [41]. In the ST dataset, the PSGs were recorded in the hospital over two nights, one of which was after temazepam intake (Temazepam night), and the other night after placebo intake (Placebo night). The PSG recordings from 2 participants were started later than their lights-off time, thus were excluded from the current study. Finally, 20 participants (7 males, 39 ± 18 years old, mean ± standard derivation) were included as the patient group from the ST dataset.

For both datasets, there were two EEG channels, i.e., Fpz/Cz and Pz/Oz, with a sample rate of 100 Hz, and only the Pz/Oz channel was used in the current study. Sleep stages were manually scored by well-trained technicians according to the Rechtschaffen and Kales rules [42] based on these EEGs. Sleep stages included wake, rapid eye movement sleep (REM) and non-rapid eye movement sleep (NREM), which consisted of sleep stage 1 (S1), stage 2 (S2), stage 3 (S3) and stage 4 (S4). The S3 and S4 stages were combined as a single stage, which was called the slow wave stage (SWS) in this study.

### 2.2. Multi-Scale Permutation Entropy Analysis

Permutation Entropy (PE) is an algorithm based on Shannon entropy proposed by Bandt et al. to measure the complexity of time series [33]. Compared with other algorithms for the evaluation of complexity, PE has the advantages of simple calculation and high robustness against noise [33]. Multi-scale Permutation Entropy (MPE), which introduces coarse-grained processing on the original time series, is generally regarded to be capable of capturing the characteristics under different time scales and making up for the shortcomings of the original PE algorithm [33].

The calculation of MPE, including a coarse-grained procedure and a calculation of PE on each coarse-grained time series, is expressed as follows.

**Figure 1:**
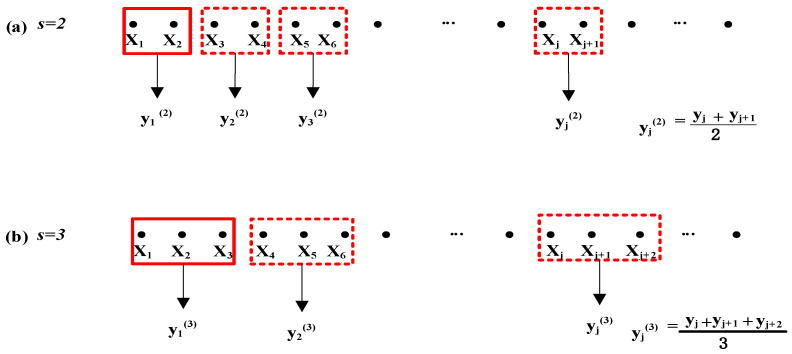
The coarse-graining process of time serie when the scale parameter equals (**a**) 2 and (**b**) 3.

For the time series $\left\{X_{1},X_{2},\ldots ,X_{N}\right\}$ of length *N*, the coarse-grained time series $\left\{y_{1}^{\left(s\right)},y_{2}^{\left(s\right)},\;\ldots ,y_{Ns}^{\left(s\right)}\right\}$ with a scale parameter *s* can be constructed by formula (1)

(1) $$y_{j}^{\left(s\right)}=\frac{1}{s}\sum_{i=\left(j-1\right)}^{js}X_{i},\;\;\;\;1\leq j\leq Ns$$

where *Ns* represents the maximal integer no more than the ratio of *N* and s. When s equals 1, the coarse-grained time series is exactly the original time series. Figure 1a,b illustrate the construction of coarse-grained time series when scale parameter s equals 2 and 3, respectively.

The phase space of the coarse-grained time series $y^{\left(s\right)}$ can then be reconstructed with an embedding dimension *m* by *Ns* − *m* + 1 vectors. The *k*th vector in this *m*-dimensional space can be described as shown in Equation (2).

(2) $$Y_{k}=\left[y_{k}^{\left(s\right)},y_{k+1}^{\left(s\right)},y_{k+2}^{\left(s\right)},\cdots ,y_{k+\left(m-1\right)}^{\left(s\right)}\right],\;\;\;\;k=1,\;2,\cdots ,Ns-\left(m-1\right)$$

**Figure 2:**
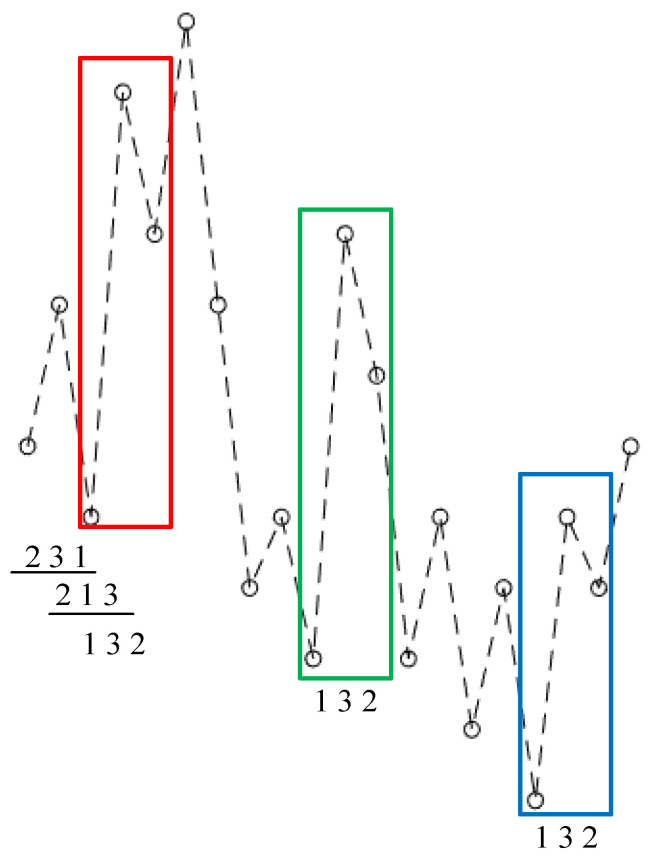
The ordinal patterns in PE calculation with an embedding dimension of 3.

Thereupon, the elements of each vector $Y_{k}$ are sorted in an ascending order to map $Y_{k}$ into an ordinal pattern, namely, a permutation $\pi_{k}$ based on the rankings of its elements. For example, a 3-dimension vector {5, 9, 2} can be mapped into {2, 3, 1}. If some elements are of equal size, they are sorted in the order as they appear. For example, a 3-dimension vector {8, 13, 8} will be mapped as {1, 3, 2}. Figure 2 illustrates how the mapping is developed with *m* = 3 on a time series with length 20.

For m-dimensional vector, it contains m elements and the number of its possible permutations equals to the factorial of m (denoted as *m*!). For each permutation $\pi_{k}$, then it is reconstructed with an embedding dimension *m* according to the Equation (2), the probability of its occurrence is calculated and shown as $p\left(\pi_{k}\right)$. Taking the time series shown in Figure 2 as an example, as the occurrence times of {1,3,2} in all the 18 vectors are 3, its probability can be regarded as 3/18. According to Shannon’s information entropy, PE can be calculated as

(3) $$\mathrm{PE}=\frac{-\sum_{k=1}^{m!}p\left(\pi_{k}\right)\log\left(p\left(\pi_{k}\right)\right)}{\log\left(m!\right)}$$

In short, PE represents the complexity of time series by considering the ordinal order of time series. Similar fluctuations are identified as the same ordinal patterns. Therefore, the stability of the whole time series can be derived by evaluating the probability of the occurrence of an ordinal pattern in a time series. The smaller the entropy value is, the more regular the time series is and the lower the complexity is. In contrast, the larger the entropy value is, the more random the time series is and the higher the complexity is.

In this paper, MPE algorithm was applied on every 30 s EEG time series for each participant. We considered the scale parameter s from 1 to 10 in steps of 1. For the reliable computation of Shannon’s information entropy, we only considered the embedding dimension *m* 3, 4 and 5 as, in the case of *m* more than 6, there are 720 or more possible permutations but only 2995 or fewer vectors for the computation of PE. The averaged MPE value of these 10 scales was taken as a single complexity measurement of each 30 s EEG series.

### 2.3. Research Framework

**Figure 3:**
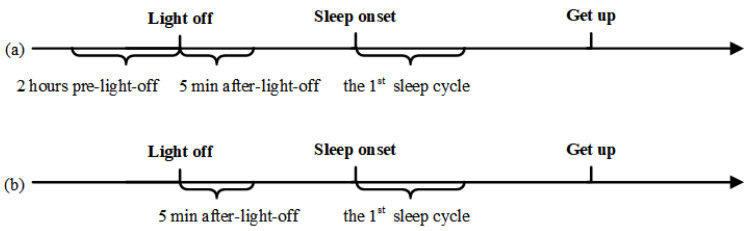
Schematic diagram of the timeline in the analyses. (**a**) The timeline for the analysis on the HC group. MPE was evaluated in three different periods, that is, 2 h pre-lights-off, 5 min after lights off, and the first sleep cycle for both nights. (**b**) The timeline for the analysis on patients with insomnia. MPE was computed over each 30 s epoch within the 5 min after lights off and the first sleep cycle for both Placebo and Temazepam night, respectively.

As shown in Figure 3a, for both nights of the HC group, we analyzed and compared the EEG complexity of the participants based on MPE algorithm before (2 h before lights off), during (5 min after lights off) and after sleep onset (the first sleep cycle); at the same time, we analyzed the MPE values of different sleep stages after sleep onset. For each period, the MPE values of all 30 s epochs were averaged as its complexity measurement.

As shown in Figure 3b, for patients with mild insomnia, we compared and analyzed the MPE during (5 min after lights off) and after sleep onset (the first sleep cycle) between first (Placebo) night and second (Temazepam) night.

There is currently no standard definition of the sleep cycles, but Feinberg I [42] sleep cycle standard is most used. Under this standard, this paper refined it as follows:

(1) Except for the first sleep cycle, each sleep cycle includes a continuous NREM and a continuous REM cycle. The first cycle does not have any requirement for REM sleep stage;
(2) For each NREM cycle in the sleep cycle, it must start from Stage 2 and last no less than 15 min. If NREM sleep is interrupted during awake stage, this will not last for over 5 min and ensure the cycle is not interrupted;
(3) The REM cycle shall be kept for more than 5 min and extended for as long as possible. The awake interruption shall not exceed 1 min.

### 2.4. Statistical Analysis

The statistical analysis in this study was performed by using MATLAB (MathWorks Inc., Natick, MA, USA) and RStudio 1.4.1717 (RStudio Inc., Boston, MA, USA). For the HC group, for each night, analysis of variance (ANOVA) with Tukey Kramer test for post-hoc was used to compare MPE values before, during and after sleep onset and to compare these values in different sleep stages. Differences of MPE between the two nights were tested by paired *t*-test for each sleep state. For patients with insomnia, paired *t*-test was used to evaluate whether the MPE significantly altered after sleep onset, compared with that during sleep transition for both Placebo night and Temazepam night, separately. Moreover, paired *t*-test was employed to compare the MPE values of Placebo night and Temazepam night during sleep transition or after sleep onset. Finally, linear regression of multiple variables was used to investigate the correlation between MPE and age as well as gender for the second night of HC group and the Temazepam night of patient group, respectively. In those models, MPE value of the 1st sleep cycle was treated as the dependent variable while age and gender were independent variables. All statistical tests were performed by two-sided test, and a *p*-value less than 0.05 was regarded as significantly different.

## 3. Results

As the test results of the embedding dimension *m* 3, 4 and 5 were similar, we only reported the results with *m* = 3 in this paper.

**Figure 4:**
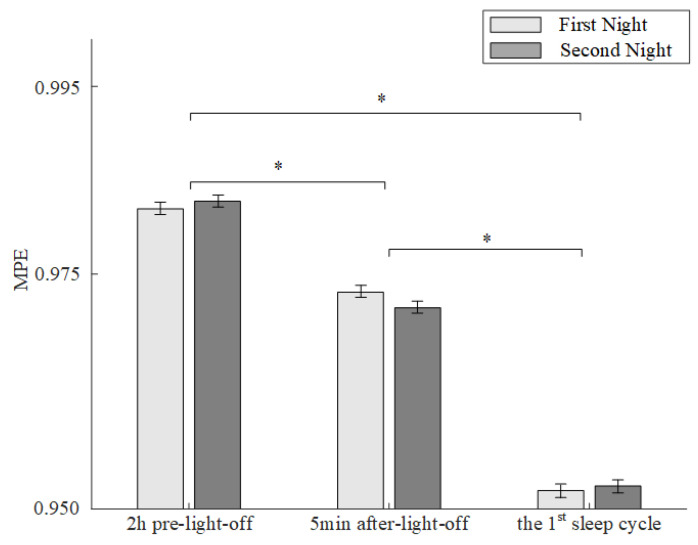
MPE Values (mean ± standard error) of HC Group before, during and after sleep onset. The symbol * represents significant differences of MPE (Tukey Kramer test, *p* < 0.05) between each two periods among 2 h pre-lights-off, 5 min after lights off and the first sleep cycle for both nights.

We analyzed and compared the averaged MPE of the HC group before, during and after sleep onset for two consecutive nights. As shown in Figure 4, for both nights, the period was the main factor that affected the MPE values (ANOVA, *p* < 0.05). Post-hoc analysis further revealed significant differences (Tukey Kramer test, *p* < 0.05) of MPE between each two states among the period of wakefulness 2 h before lights off, the sleep transition 5 min after lights off and the sleep state in the first sleep cycle. The results suggested that the MPE values decreased from the awake period to falling asleep. Moreover, comparisons between the MPE values of the HC group in the first night and the second night showed no significant differences during any periods, which suggests that no first-night effects were found in the HC group.

**Figure 5:**
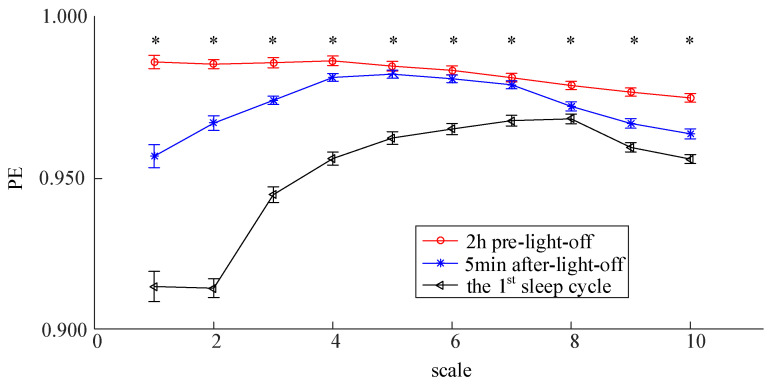
PE Values (mean ± standard error) at different scales of HC Group (in the second night) before, during and after sleep onset. The symbol * represents a main factor of period on the value of PE (ANOVA, *p* < 0.05) at the corresponding scale.

Additionally, we investigated PE differences from the awake period to the first sleep cycle at different scales for the HC group on the second night. The results are illustrated in Figure 5. A significant effect of period on PE (ANOVA, *p* < 0.05) can be observed at all ten scales. The results at most scales yielded a great similarity with that obtained using the averaged MPE, although post-hoc analysis suggests that the significant differences of PE (Tukey Kramer test, *p* < 0.05) between wakefulness and sleep transition disappeared at scales 5–7. 

**Figure 6:**
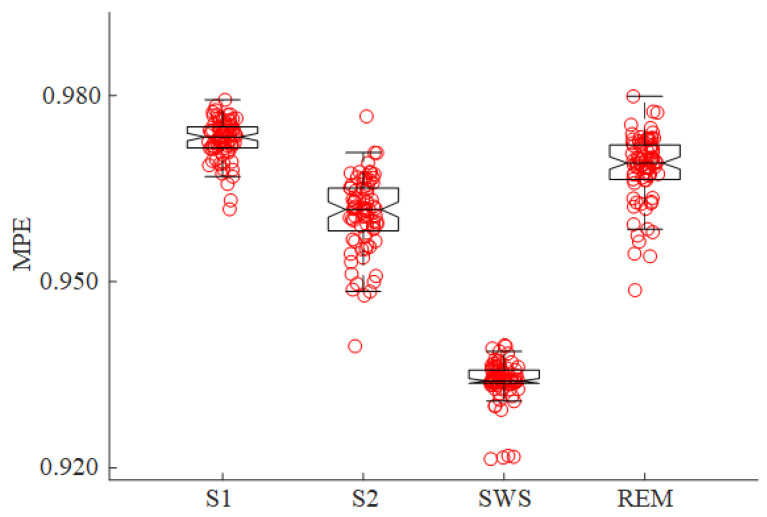
MPE values for the HC group (the second night) at different sleep stages. Each dot represents the median value of MPE for a participant during the corresponding stage. The box-plots illustrate the distribution of these median values for all participants in HC group.

For the second night, we also compared MPE values of the HC group from different sleep stages after sleep onset (as shown in Figure 6). MPE exhibited a decreasing trend from S1 to S2 and then to SWS (χ^2^ = 252.35, *p* < 0.001). The results suggest that the MPE values decreased with the deepening of sleep.

**Figure 7:**
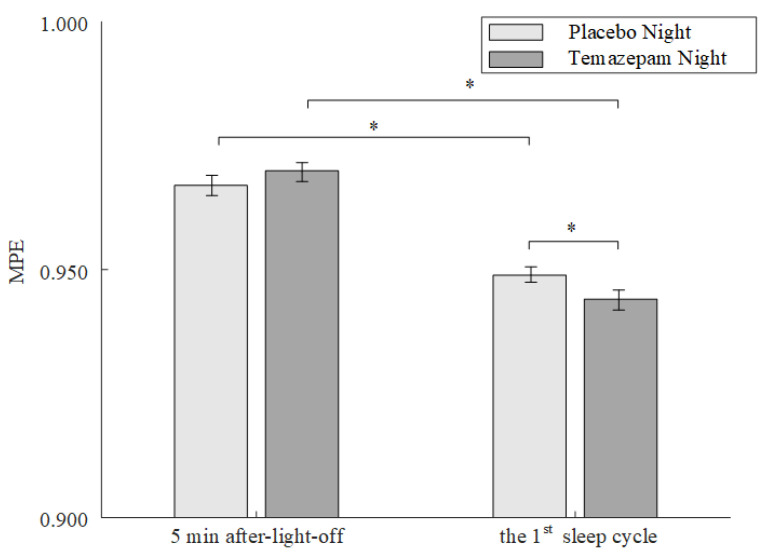
MPE values (mean ± standard error) of patients with insomnia during the 5 min after lights off and during the first sleep cycle in Placebo night and Temazepam night. The symbol * represents a significant difference of MPE between the corresponding periods in a same night (5 min after lights off or the first sleep cycle) or between both nights during a same period.

Furthermore, we compared MPE values during and after sleep onset among patients with mild insomnia. As shown in Figure 7, for both the Placebo night and the Temazepam night, the MPE after sleep onset was significantly lower than that during sleep transition (paired *t*-test, *p* < 0.05). During sleep transition, no significant differences were found in MPE between the Temazepam night and the Placebo night. However, the MPE from the Temazepam night was significantly lower than that of the Placebo night (paired *t*-test, *p* < 0.05) in the first sleep cycle.

**Figure 8:**
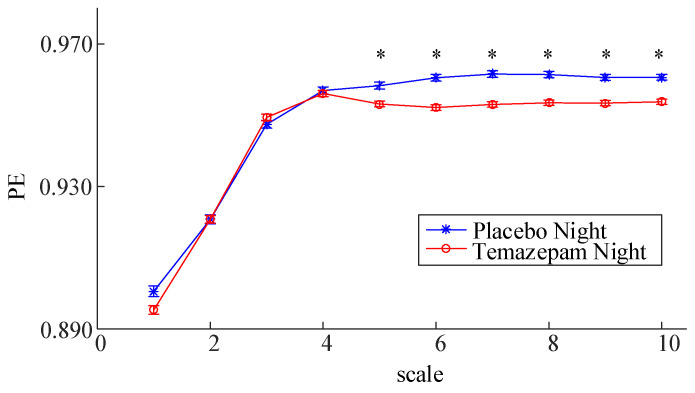
PE Values (mean ± standard error) at different scales of patient group for the Placebo night and the Temazepam night during the first sleep cycle. The symbol * represents a significant difference of PE between both nights (paired *t*-test, *p* < 0.05) at the corresponding scale.

As shown in Figure 8, for the patients with insomnia, a significant difference (paired *t*-test, *p* < 0.05) of PE between the Placebo Night and Temazepam night was only observed at larger scales (5–10) in the first sleep cycle. As larger scales corresponding to the lower frequency component in the signal, the results suggest that the improvement role in sleep quality of Temazepam might associate with its assistance in reducing the complexity of the slow wave of the EEG.

Moreover, we created a linear regression model to understand MPE in the HC group (the second night) and the insomnia group (the Temazepam night) by including age and gender. As shown in Table 1, for both groups, there was no significant correlation between MPE and gender. However, a significant correlation between age and MPE was found in the HC group but not in the insomnia patients. Our results suggest that MPE may be served as a biomarker of sedative and hypnotic effects on patients with insomnia and it may be less affected by age and gender.

## 4. Conclusions and Discussion

In this study, we investigated the role of MPE in the assessment of insomnia and temazepam treatment. Our results showed that MPE is associated with sleep depth, where the lower MPE will indicate deeper sleep stages. Among patients with insomnia, the MPE of the first sleep cycle during the Temazepam night is significantly lower than that for the Placebo night; the MPE of Temazepam night is not affected by age and gender.

Our results from healthy controls were in line with published studies. Zhang et al. analyzed the relationship between MPE and sleep stress of healthy people and suggested that lower MPE before sleep corresponded to greater sleep stress [39]. Similarly, the present results showed that the MPE of healthy people decreases from the awake period to falling asleep, which further indicates that MPE can reflect the complexity of changes in the EEG signal from the awake period to falling asleep. Our results showed that the MPE decreased from wake to stage 3 sleep, which further indicates that the decreased MPE also reflected the deeper sleep state. 

In recent years, entropy analysis of EEG signals has been widely applied in the study of many diseases, which shows that the reduced entropy can be used to diagnose early cognitive impairment, Alzheimer’s disease and high-risk infants with autism, etc. Li et al. analyzed the sample entropy of healthy subjects and patients with early cognitive impairment and found that the sample entropy of the rostral part of the anterior cingulate cortex in patients with early cognitive impairment decreased significantly [43]. Albert et al. analyzed the multi-scale entropy of EEG in patients with Alzheimer’s disease and found that the multi-scale entropy of patients with Alzheimer’s disease decreased [44,45]. Bosl et al. proved that a modified multi-scale entropy may be a biomarker of brain development. The modified multi-scale entropy of typical developing infants was higher than that of high-risk infants with autism [46]. In this paper, we found that the MPE values of patients with mild insomnia decreased significantly in the first sleep cycle during the Temazepam night compared to the Placebo night. 

Studies have proven that PSG indicators are greatly affected by age and gender, and the accuracy of evaluating the therapeutic effect of insomnia drugs was low [47,48]. This paper also analyzed PSG monitoring indicators of patients with mild insomnia, which included sleep latency, times of awakening, total sleep time, the proportion of rapid eye movement time and the proportion of slow wave sleep time. The results show that sleep latency, the number of awakenings and the proportion of light sleep in PSG monitoring indicators were related to age and gender, which was consistent with the previous research results. This paper also analyzed the correlation between MPE and age and the gender of patients with insomnia. The results showed that MPE was not associated with age and gender, which indicates that MPE was not affected by age and gender during the evaluation of drug efficacy.

In conclusion, MPE analysis of sleep EEG was found to be a potential way to measure the effect of drug efficacy in patients with insomnia. However, it should be noted that the sample size of insomnia patients in this analysis was small; further validation is encouraged on a larger sample size.

## Figures and Tables

**Table 1:** MPE with Age, Gender and Groups.

Group	Dependent Variable	Factors	*p*	Standardized Coefficient
HC (the second night)	MPE	age	0.010	0.298
		gender	0.964	0.005
Insomnia patients (the Temazepam night)	MPE	age	0.875	0.040
		gender	0.919	0.026

## Data Availability

The datasets presented in this study can be found in online repositories. The names of the repository/repositories and accession number(s) can be found below: Sleep-EDF Database Expanded: https://physionet.org/content/sleep-edfx/1.0.0/ (accessed on 20 August 2021).
